# Exploring the Negative and Gap-Widening Effects of EdTech on Young Children’s Learning Achievement: Evidence from a Longitudinal Dataset of Children in American K–3 Classrooms

**DOI:** 10.3390/ijerph19095430

**Published:** 2022-04-29

**Authors:** Jongseok Ahn

**Affiliations:** The Institute for Social Development and Policy Research, Seoul National University, Seoul 08826, Korea; ahnseok7@snu.ac.kr; Tel.: +82-10-2760-9833

**Keywords:** educational technology (EdTech), optimistic consensus on EdTech, adverse effects of EdTech, Early Childhood Longitudinal Study, Kindergarten Class of 2010-11 (ECLS-K:2011), EdTech’s effects on learning achievement, the negative effect, the gap-widening effect

## Abstract

Introducing educational technology (EdTech) into school classrooms constitutes one of the strongest educational reforms of recent decades worldwide, and as a discursive or ideological background of it, there has been the optimistic consensus on the use of EdTech among the global education community. In this context, this study highlights the dark side of EdTech and provides an opportunity for critical self-reflection of its current use through a series of quantitative analyses on a longitudinal dataset of children in K–3 American classrooms collected during the first half of the 2010s (ECLS-K:2011). In this process, two adverse effects of EdTech on young children’s learning achievement are identified: the negative effect and the gap-widening effect. These findings convey the crucial message that the education community’s approaches to EdTech should be more prudent than the current optimistic consensus. These findings do not lead us to any extreme or rigid conclusion such as techno-determinism or neo-Luddism, but rather call for a balanced and realistic deliberation on the benefits and risks of technology. This point is particularly worth clarifying in the recent situation, where schools’ dependence on EdTech has inevitably increased in response to the COVID-19 pandemic.

## 1. Introduction

### 1.1. Context and Organization of This Study

Introducing educational technology (EdTech) into school classrooms constitutes one of the strongest educational reforms in recent decades worldwide [1,2,3]. Naturally, this movement has a technological background; advances such as continuous improvement in miniaturization and performance of hardware, the development of various multimedia contents, the evolution of artificial-intelligence-based adaptive learning software, and the universalization of interactive Internet services have given rise to a variety of initiatives, including the bring-your-own-device policy, the one-laptop-per-child project, online learning approaches, and digital textbooks.

However, there has also been a discursive or ideological background that cannot be reduced to a pure technological dimension; specifically, there has been a widespread and even enthusiastic expectation among the education community that EdTech can facilitate innovations in schooling. Selwyn (2014) properly names this the “optimistic consensus” on EdTech that is prevalent among the global education community [4].

Indeed, a great amount of literature, mainly in the academic fields of education and computer industries, has represented and strengthened this mainstream thinking since the 1980s [5,6,7,8,9,10,11,12,13]. Its core messages are as follows: the various functions and contents of EdTech bring with them the opportunity to overcome the limits of standardized education or the so-called factory model of education; in other words, the prospects for pedagogical innovations, such as project-based learning (using computer functions such as simulations and presentations), hands-on learning (using multimedia contents), cooperative learning (using online communication), play-based learning (using “edutainment”-oriented educational games), and personalized learning (using the adaptive algorithms of educational software) are just around the corner; moreover, as today’s children are “digital natives” who have learned the digital language through early and daily communication with digital devices, a completely new approach to education that fully respects these characteristics is required, the key to which lies in EdTech.

In this situation, this study has been designed with the intention of highlighting the dark side of EdTech, which is generally overlooked amid the flood of mainstream thinking, and providing an opportunity for critical self-reflection on its current use in schools. By performing quantitative analyses on a longitudinal dataset collected from children in K–3 (from kindergarten to third grade) American classrooms during the first half of the 2010s, this study explores the adverse effects of EdTech on young children’s learning achievement.

The organization of this article is as follows: Section 2 introduces the materials and methods of this study. The dataset analyzed is the Early Childhood Longitudinal Study, Kindergarten Class of 2010-11 (ECLS-K:2011), which conducted a longitudinal survey on American children from the autumn of 2010, when they attended kindergarten, until the spring of 2016, when they attended fifth grade [14,15]. The variables can be categorized into three groups in terms of their roles in the models: frequency of EdTech use that the children in ECLS-K:2011 experienced in kindergarten, first grade, and second grade classrooms as independent variables; these children’s reading, math, and science achievement scores about a year later as dependent variables; and achievement scores when using EdTech as control variables as well as moderating variables.

Accordingly, Section 3 details a series of regression analyses, consequently identifying two adverse effects of EdTech. First, this study identifies the negative effect that excessive use of EdTech causes on children’s achievement scores, simply naming it “the negative effect of EdTech”. Second, this study additionally identifies the statistical interaction in the positive direction by which effects of using EdTech are differentiated in proportion to children’s achievement scores when using EdTech, naming it “the gap-widening effect of EdTech”, in the sense that it contributes to the widening of the achievement gap between low and high achievers.

The implications of these two effects are discussed in Section 4. In the process, some critical discussions of EdTech are introduced. These discussions have already warned against the potential adverse effects of EdTech since 1980s but have largely been out of the spotlight thanks to the prevailing optimistic consensus. In this context, the findings of this study imply that it is time to reappropriate the critical foresight of these discussions. The limitation of these discussions has been that they have relied too heavily on anecdotal experiences or intuitive arguments without providing sufficient quantitative evidence, and thus lack persuasiveness. This study, as an effort at evidence-based research, addresses the limitation and suggests a full review of these discussions.

Section 5 provides concluding remarks on the overall implications of this study, centered around two points: first, the findings of this study convey the crucial message that the approaches of the education community to EdTech should be more prudent than the current optimistic consensus; second, the findings do not lead us to any extreme or rigid conclusion such as techno-determinism or neo-Luddism but rather call for a balanced and realistic deliberation on the benefits and risks of technology [16]. The latter point is particularly worth clarifying in the recent situation, where schools’ dependence on EdTech has inevitably increased in response to the COVID-19 pandemic.

### 1.2. Contributions of This Study

In fact, since long before this study was conducted, there have been numerous studies that can be grouped together under the category of research on the educational effectiveness of EdTech. Thus, quantitative analyses on the effects of EdTech on elementary and secondary students’ learning achievement have been continually conducted. Nevertheless, this study differs from previous studies in at least three respects and is expected to make a significant contribution in this regard.

First, in most previous studies, EdTech has been found to have effects close to zero [2,17,18,19,20]. Considering the various monetary and non-monetary costs arising from bringing EdTech into the classroom, even such seemingly neutral results can be reinterpreted, between the lines, as having a certain degree of critical implications [2]. Still, this study goes one step further from this implicit, indirect critique. By narrowing the scope of analysis to “young children” in K–3 classrooms, this study succeeds in more clearly identifying problematic phenomena that can be fully regarded as adverse effects of EdTech.

Second, quite a few previous studies have had methodological controversies when trying to identify EdTech’s effects [3] (p. 2). The most common research design was random experiments that assign students to treatment and control groups and measure the effectiveness of using a particular part of EdTech such as educational software. However, these experiments have rarely been free from tricky issues such as the novelty effect or the Hawthorne effect [21,22,23,24]. Next, in the case of analyses of non-experimental survey datasets, there has almost always been a problem expressed in various terms such as endogeneity, omitted variable bias, and sample selection bias. Particularly at issue was the possibility of reverse causality, which arises as most of the use of EdTech in school classrooms is determined not at random but under strategic considerations of students’ prior learning achievement [25,26].

This study is another case of analysis of a non-experimental survey dataset and thus faces the same problem as described above. Nevertheless, by making use of the longitudinal structure of the dataset, the controversy is prevented to the utmost. More specifically, while children’s achievement scores measured a year later are included in the models as dependent variables, scores measured at the same time as independent variables are simultaneously included as control variables to block the possibility of reverse causality. In the field of educational effectiveness research, this type of strategy is referred to as “value-added modeling”, in the sense that the achievement gain before and after an educational intervention is identified as “value-added” [27,28].

Third, most previous studies have been satisfied with measuring the average effect of EdTech. Only a few exceptions, targeting specific cases such as the one-laptop-per-child project, have begun to explore the differentiation of effects across students of different socioeconomic status, but the research outcomes are still at a rudimentary level [29,30,31]. By taking advantage of the longitudinal structure of the dataset, this study makes progress in this direction as well: an additional analysis is conducted using achievement scores measured at the same time as independent variables as moderating variables, as well as control variables as described above; the consequent identification of the gap-widening effect of EdTech leads us to the issue of educational inequality, thus contributing to the enrichment of the EdTech debate.

## 2. Materials and Methods

### 2.1. Dataset

As briefly introduced earlier, the dataset analyzed in this study is ECLS-K:2011 provided by the National Center for Education Statistics (NCES) in the U.S. Department of Education [14,15]. The sample of this survey comprises 18,174 children who attended kindergarten across the United States during 2010–2011. Thus, focusing on their family lives, school experiences, intellectual, socioemotional, and physical development, etc., a longitudinal survey was conducted on them until 2015–2016, when they attended fifth grade, and their parents, teachers, schools, and care providers also participated in the survey. More specifically, of the whole dataset, this study analyzes the part collected up to 2013–2014, when the children attended third grade.

As the data were collected during the first half of the 2010s, they are certainly older than those collected during the second half of the 2010s or the early 2020s. Nevertheless, the timeliness can be justified by the fact that the dataset was collected in none other than the United States, one of the most advanced countries in terms of the introduction of EdTech. Since 1998, the United States has steadily implemented the “E-Rate” program that annually invests $1–2 billion with the aim of bringing EdTech into schools across the nation and through this process has become the center of the EdTech movement [1,32]. Additionally, now, many developing countries are engaged in policy efforts to catch up with the United States and other leaders in the EdTech movement under the banner of reforming public education in the 21st century [33]. Therefore, although some may point out that the dataset does not encompass the new trend in the United States in the late 2010s or the early 2020s, it has not yet lost timeliness as a dataset that appropriately reflects the latest development of EdTech.

The data file can be downloaded from the NCES website [34]. In this study, all the analyses are conducted with STATA 15.1.

### 2.2. Variables

Table 1 summarizes the variables used in this study. For readers’ convenience, serial numbers 1–20 are assigned in the leftmost column, and all the variable names are in bold letters; this style is repeated throughout this paper to make them easily recognizable. The numbers in parentheses on the right side of some variable names correspond to the time when the variables were measured: (0), (1), (2), and (3) mean that the variables were measured when children attended kindergarten, first grade, second grade, and third grade, respectively.

Nos. 1–3 in Table 1, from **frequency of EdTech use (0)** to **frequency of EdTech use (2)**, are independent variables: the frequency of EdTech use that each child in ECLS-K:2011 experienced in the kindergarten, first grade, and second grade classrooms. However, as the most suitable indicator out of the information provided by ECLS-K:2011, the frequency of using software for reading instruction is used as a proxy. Considering the substantial contribution that reading instruction makes to early graders’ curricula, this choice has sufficient validity.

The source of measurement is an item in teacher questionnaires, where teachers who managed the classrooms responded with four responses: “never or hardly ever”, “once or twice a month”, “once or twice a week”, or “almost every day”. As described in Table 1, the frequency of EdTech use seems to have remained largely unchanged throughout kindergarten, first grade, and second grade: “never or hardly ever” accounts for around 30% in each of the three variables, while “almost every day” shows roughly the same percentage.

Nos. 4–15 in Table 1, from **reading achievement score (0)** to **science achievement score (3)**, are variables that perform multiple roles as dependent, control, and moderating variables: reading, math, and science achievement scores achieved by each child while attending kindergarten, first grade, second grade, and third grade. Specifically, scores measured when children attended kindergarten are used as control and moderating variables; scores measured when children attended first grade or second grade as dependent, control, and moderating variables; scores measured when children attended third grade only as dependent variable.

The source of measurement is a series of subject-based cognitive evaluations conducted periodically on all the children, technical details of which can be found in the ECLS-K:2011 manual [15] (pp. 3.2–3.15). For each subject, the achievement scores were measured on the same scale over multiple points in time to track children’s cognitive growth. As a natural result of this, as described in Table 1, the mean values increased as children progressed to higher grades.

Nos. 16–20 in Table 1, from **household income** to **parent occupation (b)**, are additional control variables indicating children’s socioeconomic status: annual household income, highest levels of education, and current occupations of both parents of each child. All of these are categorical variables, and the numbers of their categories are described in Table 1. In order to minimize the number of observations deleted, missing values of each variable are treated as a separate category. Technical details including the complete list of the categories can be found in the ECLS-K:2011 manual [15] (pp. 7.38–7.50).

### 2.3. Models

Table 2 summarizes the two linear regression models applied in this study, which are intended to identify the negative and gap-widening effects of EdTech, respectively. Within variable names, **T** can be replaced with 0, 1, or 2 and **subject** with reading, math, or science. Thus, by applying Models 1 and 2 to nine samples produced by the different combinations of **T** and **subject**, a total of 18 regression analyses were conducted.

The purpose of Model 1 is to identify the causal effect of **frequency of EdTech use (T)**, as the independent variable, on **subject achievement score (T + 1)**, as the dependent variable. However, since **frequency of EdTech use (T)** is a categorical variable, in the actual procedure, “never or hardly ever” is treated as the reference category and three binomial variables indicating whether children fall under the three remaining categories (“once or twice a month”, “once or twice a week”, and “almost every day”) are put into the regression formulas; thus, estimations and statistical tests of their three corresponding coefficients are conducted.

However, as mentioned in Section 1.2, the use of EdTech in school classrooms is usually not determined at random. Just like all the other educational interventions, using EdTech is bound to take place under teachers’ and schools’ strategic consideration of students’ current conditions such as learning achievement, which brings us to the issue of statistical control [25]. Although most survey datasets provide information on students’ socioeconomic status, and ECLS-K:2011 is not an exception, it is widely acknowledged that this alone cannot guarantee sufficient statistical control to measure the effectiveness of any educational intervention, including EdTech [27] (p. 128). Even among students who share exactly the same socioeconomic status, learning achievement might still vary due to other factors such as intelligence, attitude, and family culture that are non-socioeconomic in nature and usually unobserved in surveys. Thus, when it comes to identifying the causal effect of an education intervention on students’ learning achievement using models only controlling for their socioeconomic status, there is a considerable possibility of reverse causality, where an unidentified part of the variation in the independent variable is conversely affected by the differences in the dependent variable induced by such non-socioeconomic, unobserved factors, thus making it troublesome to infer the existence and magnitude of the causal relationship from the very result.

To avoid this controversy, in Model 1, **subject achievement score (T)** measured at the same time as **frequency of EdTech use (T)** is introduced as a control variable. It is due to the longitudinal nature of the dataset that **subject achievement score (T + 1)** and **subject achievement score (T)** can be included together in a single model as the dependent variable and a control variable, respectively. It is expected that this approach alone prevents much of the endogeneity problem, of which reverse causality is the most representative case.

Furthermore, to eliminate any residual possibility of endogeneity other than reverse causality and to achieve the maximum statistical control that secures robust causal inferences, variables indicating the socioeconomic status of children are included as additional control variables: **household income**, **parent education (a)**, **parent education (b)**, **parent occupation (a)**, and **parent occupation (b)**. Since these are all categorical variables, in the actual procedure, binomial variables corresponding to all the categories except for the reference ones are generated and put into the regression formulas.

The purpose of Model 2 is to identify the interaction in which the effect of **frequency of EdTech use (T)** on **subject achievement score (T + 1)** is moderated by **subject achievement score (T)**. Thus, **subject achievement score (T)** now performs a double role as the moderating variable as well as a control variable, and in Model 1 where the maximum statistical control is achieved, the interaction term, **subject achievement score (T) × EdTech usage frequency (T)**, is added. However, as mentioned above, **frequency of EdTech use (T)** consists of three binomial variables in the actual procedure; thus, there are three interaction terms derived by multiplying **subject achievement score (T)** with each of those three, and estimations and statistical tests for the six coefficients are conducted.

One thing to be noticed is that a simple manipulation of the values of **subject achievement score (T + 1)** and **subject achievement score (T)** is performed before the analyses: all the values were subtracted by the mean value of each score described in Table 1, which is usually referred to as “mean centering” in the methodological literature; for example, the values of **reading achievement score (0)** is subtracted by 69.7, and so on. This manipulation has no effect on the result of statistical tests in Model 1, and in Model 2, tests for coefficient estimates of the three interaction terms are not affected either. This only prevents standard errors for coefficient estimates of the three binomial variables from becoming overinflated in Model 2.

### 2.4. Estimations and Tests

The data file of ECLS-K:2011 provides more than 100 sampling weights, and the manual recommends selecting the most appropriate one according to the range of the data analyzed. The two rationales behind this technical recommendation are minimizing the bias that missing values may cause and accounting for different sampling probabilities of observations in estimations [15] (pp. 4.28–4.52). As such, in this study, a sampling weight (W7C17P_7T170) in the data file was applied to derive the proportions, means, and standard deviations of the variables in Table 1.

On the other hand, in the estimations of coefficients in Models 1 and 2 as described in Section 3, unweighted ordinary least squares estimators are calculated. In addition, before each estimation, observations with missing values in any variables are removed, which is usually referred to as “listwise deletion” in the methodological literature. In Models 1 and 2, missing values are found in **subject achievement score (T + 1)**, **subject achievement score (T)**, and **EdTech usage frequency (T)**; thus, by removing different sets of observations with missing values according to the different combinations of **T** and **subject**, nine samples were prepared for the analyses (see Table 3).

There are three reasons for this approach. First, although the recommendation from the manual of ECLS-K:2011 is valid in principle, it should also be taken into consideration that using weighted estimators entails a certain cost. The most conspicuous weakness of weighted estimators is that they usually have larger standard deviations than unweighted ones, increasing the risk of type II errors in statistical tests. Second, since the aim of this study is not to describe the population but to identify causal relationships between specific variables, adopting an unweighted estimator is not a fatal choice. Third, in Table 3, the means and proportions of **subject achievement score (T + 1)**, **subject achievement score (T)**, and **frequency of EdTech use (T)** for each sample have been calculated after removing observations with missing values and without weighting. Nevertheless, it appears that there is no significant difference from the weighted figures in Table 2. This strongly suggests that analyses can be done without any significant bias even in the relatively simple approach of calculating unweighted ordinary least squares estimators after listwise deletions.

In the case of variance estimation to test the statistical significance of each estimate, cluster-robust standard errors that assume non-zero correlations between error terms in regression models are calculated [35]. As the cluster variable, the school identification number provided by ECLS-K:2011 can be used. However, considering that some children moved schools as years passed, in this study, three different identification numbers, assigned in the spring of 2011, 2012, and 2013, respectively, are used as cluster variables for models of different **T**s: S2_ID when **T** = 0; S4_ID when **T** = 1; and S6_ID when **T** = 2 [15] (pp. 7.4–7.7). Cluster-robust standard errors assume that the number of clusters is close to infinity. As the numbers of clusters of S2_ID, S4_ID, and S6_ID are 1308, 2091, and 2419, respectively, the assumption is satisfied quite well.

## 3. Results

### 3.1. Identifying the Negative Effect of EdTech

Table 4 shows the results of nine regression analyses that repeatedly applied Model 1 to nine samples from different combinations of **T** and **subject**. Figures in the table are estimates of the coefficients of three binomial variables derived from **frequency of EdTech use (T)**. They are differences in **subject achievement score (T + 1)** between children in one of the three categories (“once or twice a month”, “once or twice a week”, or “almost every day”) on the one hand and those in the reference category (“never or hardly ever”) on the other, under the condition that those two groups share equal values in control variables including **subject achievement score (T)**. In a nutshell, they are relative gains or losses by children in one of the three categories compared to those in the reference category.

Of the 27 estimates, shading has been added to statistically significant ones that have *p*-values less than 0.05, for readers’ convenience. On the whole, there are four statistically significant estimates, all of which show negative signs. In other words, only relative losses have been identified, without any case of relative gains.

Interestingly, as **T** decreases from 2 to 0, more statistically significant estimates are identified. First of all, when **T** = 2, all estimates are statistically insignificant. In other words, regardless of which one of the four categories of **frequency of EdTech use (2)** children belong to, they do not show any meaningful difference in **reading achievement score (3)**, **math achievement score (3)**, or **science achievement score (3)**.

On the other hand, the problem begins to appear when **T** = 1. While children in the “once or twice a month” or the “once or twice a week” category of **frequency of EdTech use (1)** still show statistically insignificant differences in all scores compared to children in “never or hardly ever”, children in “almost every day” eventually show a relative loss in **reading achievement score (2)**.

The problem becomes clearer when **T** = 0. Children in the “once or twice a week” category of the **frequency of EdTech use (0)** already show a relative loss in the **math achievement score (1)**. Further, children in “almost every day” show an additional loss in **reading achievement score (1)** while showing a larger loss in the **math achievement score (1)** than those in “once or twice a week”.

As explained earlier, Model 1 succeeds in achieving the maximum statistical control due to the longitudinal structure of the dataset. As such, the result in Table 4 indicates quite robustly the causal relationship between the excessive use of EdTech in the classroom and children’s lowering achievement scores—beyond a mere correlation. In that sense, it can be considered as evidence for a potential adverse effect of EdTech on young children’s learning achievement. As already mentioned, this study simply names it “the negative effect of EdTech”.

### 3.2. Identifying the Gap-Widening Effect of EdTech

Table 5 presents the result of repeatedly applying Model 2 to nine samples. This time, together with the coefficients of the three binomial variables from **frequency of EdTech use (T)**, the coefficients of the three interaction terms derived by multiplying **subject achievement score (T)** with those three are estimated. As in Table 4, shading has been added to statistically significant estimates.

First of all, in the case of 27 estimates for the coefficients of the three binomial variables, exactly the same ones as in Table 4 are statistically significant and negative. In addition, in terms of figures, there are only minute changes in the second digit after the decimal point. In other words, the negative effect of EdTech identified in Table 4 has been reaffirmed.

Next, of the 27 estimates for the coefficients of the three interaction terms, there are three statistically significant ones. Moreover, all of them show positive signs, which means that relative gains or losses by children in a category of **frequency of EdTech use (T)** are moderated in the positive direction by **subject achievement score (T)**; thus, each child achieves an additional gain or loss proportionate to his or her **subject achievement score (T)**.

Interestingly, this time, as **T** increases from 0 to 2, more statistically significant estimates are identified. First, when **T** = 0, no interaction is statistically significant. In other words, the effects of **frequency of EdTech use (0)** on **reading achievement score (1)**, **math achievement score (1)**, and **science achievement score (1)** are uniform between low and high achievers.

On the other hand, the problem begins to appear when **T** = 1. The relative gain or loss in **reading achievement score (2)** by children in the “once or twice a week” category of the **frequency of EdTech use (1)** compared to those in “never or hardly ever” is moderated in the positive direction by **reading achievement score (1)**.

The problem becomes clearer when **T** = 2. Relative gains or losses in **reading achievement score (3)** and **math achievement score (3)** made by children in the “almost every day” category of **frequency of EdTech use (2)** are moderated in the positive direction by **reading achievement score (2)** and **math achievement score (2)**, respectively.

The specific meaning of “moderated in the positive direction” is as follows: for example, in the first case among the three statistically significant estimates aforementioned, the main effect of the binomial variable corresponding to the “once or twice a week” category of **use of EdTech frequency (1)** on **reading achievement score (2)** is −0.42; however, since the value is statistically insignificant, it can be considered practically zero, and what remains is the interaction effect, (0.03) × **reading achievement score (1)**; as **reading achievement score (1)** has been mean-centered, its adjusted mean is now zero, with a standard deviation (SD) of 17.59 (see Table 1); therefore, additional gains or losses by children located at −2SD, −1SD, 0, +1SD, +2SD on the distribution of **reading achievement score (1)** are −35.18, −17.59, 0, +17.59, and +35.18, respectively. The second and third cases can be understood in the same way.

As in Model 1, the maximum statistical control is achieved in Model 2. Thus, another causal inference can be drawn regarding the results shown in Table 5. Due to the existence of the interaction in the positive direction, children achieving above the mean when using EdTech achieve additional gains from it, while children achieving below the mean suffer additional losses; as a result, the use of EdTech contributes to the widening of the achievement gap between high and low achievers. This can be justly considered as evidence of another type of adverse effect of EdTech on young children’s learning achievement. As already mentioned, this study names it “the gap-widening effect of EdTech”.

### 3.3. Two Interesting Patterns

Meanwhile, two interesting patterns were found in Table 4 and Table 5, drawing our attention. Their implications are briefly discussed in Section 4.3.

First, the negative and gap-widening effects of EdTech were identified in reading and math achievement scores, and it is somewhat difficult to determine in which subject the effects are stronger. On the other hand, when it comes to science achievement scores, no statistically significant coefficient estimates related to either effect have been identified.

Second, as pointed out briefly in Section 3.1 and Section 3.2, although problematic tendencies that can be referred to as the negative and gap-widening effects of EdTech do make their appearance in Table 4 and Table 5, respectively, they do not appear uniformly at all points in time when the frequency of EdTech use is measured. As **T** increases from 0 to 2, the negative effect gradually weakens and even disappears, while the gap-widening effect instead strengthens.

## 4. Discussion

### 4.1. Implication of the Negative Effect of EdTech

The critical implication of this study is clear due to the identification of the negative effect of EdTech. There is little doubt that such an effect is a serious challenge to one of the primary goals of schooling: promoting intellectual growth of children by providing the best opportunities to learn. In this sense, the existence of the negative effect can be rightly regarded as strong evidence that necessitates a fundamental reconsideration of the optimistic consensus on EdTech among the global education community.

In fact, not all discussions so far have been part of the optimistic consensus. Since the 1980s, there have been critical discussions steadily warning of the potential adverse effects of EdTech, although only by a relatively small number of writers and speakers [36,37,38,39,40,41,42,43,44]. Nevertheless, as pointed out in Section 1.1, the biggest limitation of the literature has been its failure to provide sufficient quantitative evidence to support such critical discussions. The findings of this study may have the impact of allowing for the rediscovery of the insights in the literature while also providing evidence to compensate for such a limitation.

The main grounds for the optimistic consensus on EdTech, without any positive or negative evaluations, can be described as follows [5,6,7,8,9,10,11,12,13]:

- In contrast to the traditional classroom, where learning has relied almost excessively on texts, multimedia content such as images, videos, and music allows for vivid hands-on learning by stimulating the visual, auditory, and kinesthetic senses. In particular, with immersive technologies such as virtual and augmented reality being developed nowadays, such an advantage has been greatly enhanced.
- The internet enables students to search for relevant information and knowledge as quickly as possible and to communicate in real time with teachers and peers whenever they want. Moreover, by utilizing functions on the computer, the result of such activities can be expressed in a variety of presentation formats. This whole process encourages project-based, self-directed learning and promotes students’ creativity.
- “Edutainment”-oriented educational software that incorporates the framework of computer games into learning transforms traditional, rigid classes into easier and more pleasant ones.
- Artificial-intelligence-based adaptive software provides personalized learning that is consistent with the ability, attitude, and academic progress of each child.
- Today’s children, as digital natives, show unprecedented strengths in information search, visualization, gaming, networking, etc., compared to older generations. Introducing EdTech has the meaning of reforming education in a direction more suitable to the characteristics of the new generation.

On the contrary, critical discussions have presented at least partial disagreements and even all-out counterarguments against each of the above grounds, as follows [36,37,38,39,40,41,42,43,44]:

- Excessive dependence on multimedia content and sensory stimuli therefrom often damages the scope, sequence, and balance of the school curriculum, and such a dumbing down of schooling adversely affects students’ cultivation of basic skills such as literacy and numeracy.
- The information that students receive from the Internet is often inaccurate and unverified, and computer-mediated presentations are frequently limited to shallow, cut-and-paste works combined with students’ limited background knowledge. These realities are far from the ideal of self-directed learning and creativity.
- The idea of “edutainment” promotes the misunderstanding that learning is entertainment itself, thus sometimes rendering it unsuitable for cultivating the virtues necessary for learners, such as patience and concentration. Furthermore, activities unrelated to learning such as games, chatting, and Internet surfing are not that easily controlled. These lead to the problem of distraction and do harm to the classroom atmosphere.
- Overheated expectations for personalized learning disparage the importance of the standardized curriculum containing core knowledge that must be completed in each grade, resulting in gaps in learning.
- Although it is undeniable that today’s children are relatively familiar with the use of digital devices, glorifying the introduction of EdTech based only on such a reality can be a populist approach overlooking the responsibility of schooling. In particular, considering many anti-intellectual problems induced by unrestrained cyber activities of children and adolescents (e.g., the decline of reading, limited vocabulary, avoidance of analytical thinking, lack of attention), such an approach can be just like adding fuel to the fire.

As the optimistic consensus on EdTech has been established as the mainstream thinking over the past decades, these critical discussions seem to have usually been dismissed as anachronistic skepticism or rigid traditionalism lacking imagination. In such a situation, this study’s finding of the negative effect of EdTech implies that it is time to listen closely again to the warnings of the critical discussions on it—at least as to its use in classrooms of young children below a certain grade or age. For now, the above five points seem to present quite convincingly and also comprehensively the latent factors driving the negative effect of EdTech.

### 4.2. Implication of the Gap-Widening Effect of EdTech

The identification of the gap-widening effect of EdTech doubles the critical implication of this study. This leads us to another ominous possibility: the misuse of EdTech is a factor that deepens the problem of educational inequality.

What is illuminated is this study is an indicator of educational inequality: the achievement gap between low and high achievers [45]. In other words, it is the variability of the distribution of students’ learning achievement. To be sure, the existence of the gap or variability itself is not a problem. Rather, considering that all learners have their own personal traits such as intelligence and aptitude, it is inconceivable that every student will record perfectly uniform scores in the learning achievement.

Nevertheless, it would be desirable at least to keep the gap below a certain threshold and furthermore to gradually narrow the existing gap through stronger support for low achievers. This is especially true in that much of the gap has been determined by structural factors such as socioeconomic status, regardless of each student’s personal traits. This unpleasant reality has established itself as a stylized fact in the field of sociology of education since the publication of the Coleman Report in the United States in 1966, and unfortunately, it has been reverified through numerous studies over the past half century [46,47,48].

Therefore, it is highly likely that a significant—or even absolute—proportion of low achievers have come to belong to the disadvantaged positions due to structural factors beyond their personal efforts and will. In this situation, even if it is impossible to completely close the gap, gradually narrowing it creates the possibility of reducing the space where such socioeconomic factors exert their unfair influences, consequently contributing to the enhancement of educational equality; on the other hand, widening it means distorting the distribution of learning achievement toward a state more vulnerable to those factors, thereby worsening the problem of educational inequality. Unfortunately, according to the findings of this study, the use of EdTech is a case of the latter.

Section 4.1, focusing on the universal effects of EdTech on all students, has already pointed out several main grounds for the optimistic consensus on EdTech. However, in addition to those grounds, there has been a supplementary one that has helped the optimistic consensus to make a stronger appeal and thus to take root deeper in the education community: the expectation that EdTech will make schooling a more equal, fair, and democratic process [49] (pp. 26–41). There are two detailed reasons behind this claim:

- EdTech is a very attractive teaching tool in terms of its efficiency and has the potential to be a powerful catalyst to resolve educational inequality. In particular, its efficient components such as smart devices, open-source software, and online open courses can boost access to education for students who are under socioeconomically disadvantaged conditions and thus suffer considerable deprivation in various tangible and intangible educational resources.
- The traditional style of teaching, in which a teacher unilaterally transmits textbook content to students and imposes rote learning through repeated practice and memorization, has alienated many children who have failed to adapt to it. In particular, students who do not possess enough cultural capital to succeed in such a traditional style have been at the center of such alienation. On the contrary, EdTech’s various advantages listed in Section 4.1 (enabling hands-on learning with multimedia content, relaxation of rigid classes, etc.) ensure more diversified learning opportunities to those who have been alienated so far and encourage them again to participate.

On the other hand, closely connected with the issues introduced in Section 4.1 are the following disagreements and counterarguments to each of the above two reasons, which critical discussions on EdTech have presented [49] (pp. 41–53):

- EdTech’s efficiency has been exaggerated compared to its actual outcomes, and furthermore, the idea of resolving educational inequality by universalizing access to education through EdTech seems to be a scenario far from reality. In the case of open online courses, for example, it appears that it is not that easy to maintain students’ motivation to participate compared to offline courses. Moreover, even if there are students who do participate well in online courses, most of them rather appear to be high-achieving—and socioeconomically advantaged—ones.
- What have been praised as EdTech’s strengths rather appear to be causing various adverse effects such as dumbing down of school curricula, decline in students’ literacy and numeracy, promotion of distraction in classrooms, etc. (see Section 4.1). The impact of these side effects is likely to be even stronger for low-achieving children who have not yet developed adequate basic skills, or socioeconomically disadvantaged ones who lack sufficient learning opportunities out of school. Oppenheimer (2003), one of the leading critics in the late 1990s and early 2000, summarized this problem in the following acrimonious phrase: “fooling the poor with computers” [39] (pp. 62–95).

This study’s finding of the gap-widening effect of EdTech suggests that the critical discussions be seriously reviewed regarding the issue of educational inequality as well—together with the critical points introduced in Section 4.1. Following the negative effect of EdTech, the gap-widening effect should be regarded as another challenge to a primary goal of schooling: functioning as a great equalizer. Twentieth-century American sociologist James S. Coleman, well-known as the first author of the Coleman Report, properly summarized it in the following quote: “The effectiveness of the school consists, in part, of making the conditional probabilities less conditional—that is, less dependent on social origins” [50] (pp. 122–123). In addition, as explained above, insofar as widening of the achievement gap between low and high achievers is a grave signal of the deterioration of the educational inequality, the gap-widening effect can be rephrased as “the inequality-worsening effect”.

Before finishing this subsection, it is worth mentioning three additional points. First, the implications derived from Section 4.1 and Section 4.2 need to be considered in an integrated manner. The coexistence of the negative and gap-widening effects is no less than an overall threat to the two primary goals of schooling—enhancing intellectual growth and ensuring equal opportunity. Borrowing the usual terms from the education community, both can simply be referred to with two single words: excellence and equality [51]. In fact, whether the two goals can be met at the same time or whether there is a potential trade-off between these two has been one of the classical questions surrounding the prospect of modern schooling [52]. In this respect, the coexistence of the two adverse effects of EdTech is a paradoxical counterevidence against the potential trade-off—as a case where excellence and equality are threatened at the same time.

Second, if only the negative effect of EdTech had been identified, the following doubt may have been raised: Is it valid to evaluate EdTech’s effectiveness in children’s learning achievement solely with subject-based test scores and to draw the conclusion that EdTech is causing the negative effect? Is there not a possibility that new positive outcomes not measurable by traditional scales—such as “21st century digital skills” [53]—emerge at the same time, neutralizing the negative effect or even reversing it? This issue, regarding the external validity of the dependent variable, does point to a limit of this study and requires consideration. Nevertheless, even if such a doubt is assumed to be reasonable to a certain extent and the critical implication of the negative effect of EdTech is somewhat alleviated accordingly, the coexistence of the gap-widening effect causes another level of problem that cannot be resolved by this assumption: unfair divergence of the EdTech effect by which high achievers enjoy additional gains and low achievers suffer additional losses. One of the original contributions of this study introduced in Section 1.2—the enrichment of the EdTech debate through the exploration of the differentiation effects across students of different status—can be appreciated in this context.

Third, the implication of the gap-widening effect is also worth considering in relation to the recent evolution of a well-known digital-sociological concept: digital divide. As explained by van Dijk (2020) [54], since its first appearance in 1995, the concept has expanded to three levels of meaning [3] (p. 5). First, until the early 2000s, the concept remained at the first level: a gap in the physical access to computers, the Internet, and other digital resources among different socioeconomic categories (class, race, age, gender, etc.). However, as digital resources gradually became universal throughout society, the center of gravity shifted to higher levels. Thus, since the mid-2000s, the second level has risen as a new understanding of the concept: a gap in the skill of use of digital resources. Then, since the early 2010s, the third level has come to gain attention: a gap in the outcomes from using digital resources.

Although the digital divide problem is an issue encompassing all of society, discussions on education have always been an integral part of it [3] (p. 5). The finding of this study is expected to make a similar contribution, as the existence of the gap-widening effect of EdTech can be regarded as a newly found case of the deterioration of the third-level digital divide—in the sense that school outcomes from EdTech appear to be unequally distributed between relatively advantageous and disadvantageous students. This reality is quite paradoxical considering the fact that one of rationales that justify the massive investment in EdTech has been bridging the first-level digital divide between socioeconomically advantaged and disadvantaged children [55].

### 4.3. On the Two Interesting Patterns

Meanwhile, as mentioned in Section 3.3, two interesting patterns in Table 4 and Table 5 need to be discussed.

First, the pattern that the negative and gap-widening effects are identified in reading and math achievement scores while not in science scores strongly implies that the EdTech effect may vary depending on the subjects. Once the results of Table 4 and Table 5 are to be accepted as reflecting a general tendency, adverse effects of EdTech on young children’s learning achievement seem to be strongest in the most basic subjects, where skills such as literacy and numeracy are to be trained, while being mitigated to some extent or even neutralized in subjects of a more applied nature such as science, history, social studies, etc. In addition, in the process, some beneficial effects of EdTech might exist in the achievement of such applied subjects, offsetting any adverse effects on literacy or numeracy. If so, at least in some applied subjects, some promises of the optimistic consensus introduced in Section 4.1 and Section 4.2 might become realized to a certain degree.

Second, the pattern that the negative effect gradually weakens while the gap-widening effect strengthens as **T** increases from 0 to 2 needs to be interpreted with more subtlety than the former one. If only the result of Table 4 is to be considered, it is certainly a welcome phenomenon that the later EdTech is used the weaker its negative effect becomes; it can be interpreted as an encouraging signal that the negative effect is a problem only for very young children who lack self-control to prevent overindulgence in computer-related activities. However, as the result of Table 5 must be put together into the whole picture, the situation turns out to be less encouraging; as EdTech is used in higher grades, the adverse effects appear to change their dominant form rather than disappear—from the negative effect to the gap-widening effect. While the former is identified relatively easily and clearly as a direct effect emanating from EdTech, the latter is more invisible and harder to identify as an effect through an indirect path; in other words, the mechanism of the adverse effects of EdTech on young children might become more sophisticated as it is used in higher grades.

## 5. Conclusions

From the discussions so far, the key message of the findings of this study becomes evident: the educational community’s approach to EdTech should be more prudent than the current optimistic consensus, which requires a thorough self-reflection on its current use in schools. In this process, it may be necessary to raise some quite uncomfortable but relevant questions as follows: Has the introduction of EdTech so far been based on purely educational, child-centered considerations? Have there not been any non-educational—or even anti-educational—factors such as commercial interests of EdTech suppliers [3] (p. 17)?

To be sure, the findings of this study and their critical implications do not lead us to any extreme or rigid conclusion, such as techno-determinism that wrongly assumes the effects of technology as immutable, or—as a corollary of such deterministic thinking—neo-Luddism that insists on the complete removal of technology from school classrooms [3] (p. 17). In this regard, from the analyses and discussions in the previous sections, at least two points are worth recalling. First, as stated from the title on, the focus of this study has been narrowed to young children in K–3 classrooms; thus, it remains an open question whether similar adverse effects would be identified among students in higher grades (e.g., high school or college students) [56]. Second, as pointed out in Section 4.3, adverse effects of EdTech seem to be conspicuous in basic subjects such as reading and math, whereas these are mitigated or neutralized in applied subjects such as science; in other words, the specific way and context in which EdTech is used in each subject seem to matter.

In addition, it is worth pondering over the implications of the statistical interaction named the gap-widening effect. As a sign of deepening educational inequality due to the misuse of EdTech, the identification of such an effect itself is clearly an unpleasant phenomenon. However, seen from a different angle, it also means that the effectiveness of EdTech depends on the capabilities of human users—such as students’ learning ability when they use EdTech.

Thus, the findings of this study should be taken as a call for a balanced and realistic deliberation on the benefits and risks of technology. This point is particularly worth clarifying in the recent situation where schools’ dependence on EdTech has inevitably increased to a considerable degree in response to the COVID-19 pandemic, mainly in the form of web-based distance learning. Besides, just in time, discussions in line with the abovementioned conclusion have recently begun to emerge globally.

For example, OECD (2020), while acknowledging the usefulness of technology as one of “the right policy tools for education delivery when schools may be closed, require social distancing or hybrid measures to respond to health needs”, does not forget to remind us of the dark side of it either: “However, digital use has also been associated with detrimental effects on students’ health and well-being. The literature has highlighted, among others, negative effects on sleep, attention, and learning; a higher incidence of obesity and depression; and exposure to inaccurate, inappropriate, or unsafe content and contacts…. Any digital strategy should take into account these potential risks, and balance digital use with screen-free activities” [57] (pp. 27–28).

A more explicitly critical viewpoint can be found in Hargreaves (2020) [44]. He expresses deep concern on “a new mantra” spreading in the midst of the pandemic crisis: “reimagining education” with digital technology. To be sure, he does appreciate the role of “an invaluable stopgap” that technology has played during the pandemic. Nevertheless, as he makes it clear, “if necessity is the mother of invention, we should also avoid making a virtue out of a necessity”. His vision of “the education technology we will and won’t need after the pandemic” is as follows: “When they get back to school, children will not need more of the anytime-anywhere Big Tech strategy…. We can benefit from using digital technology in learning. But we need to do it in a way that deliberately uses technology in a balanced (not just a hybrid or blended) way, and that maximizes the benefits, while minimizing the clear risks of excess screen time and digital addiction”.

To realize this vision, Hargreaves proposes a rule that sounds quite conservative. “A balanced approach to digital technology use should also pinpoint areas where it uniquely provides something of value that cannot be offered in any other way. This is what the business field calls its ‘unique value proposition’ (UVP). One UVP of digital technology occurs when children with special needs are given devices and programs to access and express their learning. Another is when teachers in small, remote rural schools can connect with and learn from colleagues in their subject or grade level who teach elsewhere. These are just two of the many UVPs of digital technology use in schools…. It’s not just hybrids or blends we want. We need a thoughtful balance that uses the UVP of technology wherever it can improve learning and well-being, while actively avoiding excess screen time that might disturb that balance, and continuing to promote outstanding face-to-face teachers and teaching that are still the cornerstone of an effective school system”.

As is evident in the above quotation, Hargreaves’ fundamental premise is that technology “should not be the main driver or leverage for reimagining better learning in schools”. This may sound unpleasant or even offensive to educationalists who are faithful to the optimistic consensus, or to EdTech suppliers. Nevertheless, the findings of this study suggest that such a conservative approach may in fact be a breakthrough for the sustainable use of EdTech in the long run.

To be sure, the findings of this study have certain limitations, which should be supplemented by future studies. Two representative ones are as follows. First, although the timeliness of the dataset used in this study is justified in Section 2.1, it would certainly have been better if more recent one could be obtained and analyzed. If new datasets that cover more up-to-date periods and have good quality comparable to that of ECLS:K-2011 become available, follow-up studies and comparisons with this study’s findings should be carried out. Second, as has been stressed throughout, this study strategically narrowed the focus to young children in K–3 classrooms. Thus, as pointed out above in this subsection, it is still an open question whether similar adverse effects would also be found among older students—which stimulates the need for future research aimed at more diverse education levels.

## Figures and Tables

**Table 1:** Variables.

No.	Name	Role	Definition	Description	
1	**Frequency of** **EdTech use (0)**	IV	Frequency of using software for reading instruction in the classroom that each child experienced while attending kindergarten Measured in the spring of 2011	Never or hardly ever	27.2%
				Once or twice a month	12.4%
				Once or twice a week	28.3%
				Almost every day	32.1%
2	**Frequency of** **EdTech use (1)**	IV	Frequency of using software for reading instruction in the classroom that each child experienced while attending first grade Measured in the spring of 2012	Never or hardly ever	32.0%
				Once or twice a month	16.4%
				Once or twice a week	26.0%
				Almost every day	25.7%
3	**Frequency of** **EdTech use (2)**	IV	Frequency of using software for reading instruction in the classroom that each child experienced while attending second grade Measured in the spring of 2013	Never or hardly ever	32.4%
				Once or twice a month	16.7%
				Once or twice a week	27.2%
				Almost every day	23.7%
4	**Reading achievement score (0)**	CV, MV	Reading achievement score achieved by each child while attending kindergarten Measured in the spring of 2011	Mean	69.7
				Std. dev.	14.4
5	**Reading achievement score (1)**	DV, CV, MV	Reading achievement score achieved by each child while attending first grade Measured in the spring of 2012	Mean	95.6
				Std. dev.	17.6
6	**Reading achievement score (2)**	DV, CV, MV	Reading achievement score achieved by each child while attending second grade Measured in the spring of 2013	Mean	113.0
				Std. dev.	16.6
7	**Reading achievement score (3)**	DV	Reading achievement score achieved by each child while attending third grade Measured in the spring of 2014	Mean	121.6
				Std. dev.	15.0
8	**Math achievement score (0)**	CV, MV	Math achievement score achieved by each child while attending kindergarten Measured in the spring of 2011	Mean	50.7
				Std. dev.	13.2
9	**Math achievement score (1)**	DV, CV, MV	Math achievement score achieved by each child while attending first grade Measured in the spring of 2012	Mean	73.3
				Std. dev.	15.4
10	**Math achievement score (2)**	DV, CV, MV	Math achievement score achieved by each child while attending second grade Measured in the spring of 2013	Mean	90.8
				Std. dev.	17.8
11	**Math achievement score (3)**	DV	Math achievement score achieved by each child while attending third grade Measured in the spring of 2014	Mean	104.8
				Std. dev.	17.5
12	**Science achievement score (0)**	CV, MV	Science achievement score achieved by each child while attending kindergarten Measured in the spring of 2011	Mean	34.2
				Std. dev.	7.4
13	**Science achievement score (1)**	DV, CV, MV	Science achievement score achieved by each child while attending first grade Measured in the spring of 2012	Mean	43.4
				Std. dev.	10.3
14	**Science achievement score (2)**	DV, CV, MV	Science achievement score achieved by each child while attending second grade Measured in the spring of 2013	Mean	52.9
				Std. dev.	11.6
15	**Science achievement score (3)**	DV	Science achievement score achieved by each child while attending third grade Measured in the spring of 2014	Mean	60.4
				Std. dev.	11.8
16	**Household income**	CV	Annual household income of parents of each child Measured in the spring of 2011	18 categories, from “$5000 or less” to “$200,001 or more” With an additional category for missing values	
17	**Parent education (a)**	CV	Highest level of education completed by a parent of each child designated as Parent 1 in ECLS-K:2011 Measured in the autumn of 2010 and the spring of 2011	Eight categories, from “eighth grade or below” to “master’s degree (MA, MS) or higher” With an additional category for missing values	
18	**Parent education (b)**	CV	Highest level of education completed by another parent of each child designated as Parent 2 in ECLS-K:2011 Measured in the autumn of 2010 and the spring of 2011	Eight categories, from “eighth grade or below” to “master’s degree (MA, MS) or higher” With an additional category for missing values	
19	**Parent occupation (a)**	CV	Occupation of a parent of each child designated as Parent 1 in ECLS-K:2011 Measured in the autumn of 2010	22 categories, based on the industry and occupation codes used in ECLS-K:2011 With an additional category for missing values	
20	**Parent occupation (b)**	CV	Occupation of another parent of each child designated as Parent 2 in ECLS-K:2011 Measured in the autumn of 2010	22 categories, based on the industry and occupation codes used in ECLS-K:2011 With an additional category for missing values	

IV: independent variable; DV: dependent variable; CV: control variable; MV: moderating variable. Proportions, means, and standard deviations have been weighted by a sampling weight (W7C17P_7T170).

**Table 2:** Models.

Model	Specification
1	Linear regression of **subject achievement score (T + 1)** on **frequency of EdTech use (T)**, controlling for **subject achievement score (T)** together with **household income, parent education (a)**, **parent education (b)**, **parent occupation (a)**, and **parent occupation (b)**
2	Linear regression of **subject achievement score (T + 1)** on **frequency of EdTech use (T)** and **subject achievement score (T) × frequency of EdTech use (T)**, controlling for **subject achievement score (T)** together with **household income, parent education (a)**, **parent education (b)**, **parent occupation (a)**, and **parent occupation (b)**

Within variable names, **T** can be replaced with 0, 1, or 2 and **subject** with reading, math, or science. All the values of **subject achievement score (T + 1)** and **subject achievement score (T)** are mean-centered based on the mean value of each score described in Table 1.

**Table 3:** Samples.

T	Subject	Sample Size	Sample Mean of Subject Achievement Score (T + 1)	Sample Mean of Subject Achievement Score (T)	Sample Proportions of Frequency of EdTech Use (T)			
					(a)	(b)	(c)	(d)
**0**	**Reading**	13,592	94.6	69.2	27.4%	12.5%	29.0%	31.1%
	**Math**	13,554	72.4	50.2	27.4%	12.5%	28.9%	31.2%
	**Science**	13,389	42.7	33.7	27.5%	12.6%	28.8%	31.1%
**1**	**Reading**	11,922	112.9	95.6	31.8%	16.5%	25.6%	26.1%
	**Math**	11,918	90.7	73.3	31.8%	16.5%	25.7%	26.1%
	**Science**	11,891	52.6	42.9	31.8%	16.5%	25.7%	26.0%
**2**	**Reading**	11,219	120.9	112.3	32.6%	16.7%	26.2%	24.6%
	**Math**	11,221	103.9	90.1	32.6%	16.7%	26.2%	24.5%
	**Science**	11,209	59.8	52.3	32.6%	16.7%	26.1%	24.5%

Unlike the figures in Table 2, sample means and proportions were calculated without weighting.

**Table 4:** Estimates of the coefficients of **frequency of EdTech use (T)** in Model 1.

T	Subject	Estimates of the Coefficients of Frequency of EdTech Use (T) (“Never or Hardly Ever” as the Reference Category)		
		Once or Twice a Month (Yes = 1, No = 0)	Once or Twice a Week (Yes = 1, No = 0)	Almost Every Day (Yes = 1, No = 0)
**0**	**Reading**	0.47	−0.04	−0.75 *
	**Math**	0.00	−0.05 *	−0.91 **
	**Science**	−0.03	−0.12	0.04
**1**	**Reading**	0.23	−0.42	−0.49 *
	**Math**	0.33	−0.09	−0.39
	**Science**	0.10	−0.17	−0.02
**2**	**Reading**	−0.07	−0.26	−0.08
	**Math**	0.26	−0.04	−0.07
	**Science**	−0.05	−0.14	−0.14

** *p* < 0.01; * *p* < 0.05. Shading has been added to statistically significant estimates. Cluster-robust standard errors are calculated with the cluster variable S2_ID when **T** = 0; S4_ID when **T** = 1; and S6_ID when **T** = 2.

**Table 5:** Estimates of the coefficients of **frequency of EdTech use (T)** and **subject achievement score (T)** × **frequency of EdTech use (T)** in Model 2.

T	Subject	Estimates of the Coefficients of Frequency of EdTech Use (T)			Estimates of the Coefficients of Subject Achievement Score (T) × Frequency of EdTech Use (T)		
		Once or Twice a Month	Once or Twice a Week	Almost Every Day	Subject Achievement Score (T) × Once or Twice a Month	Subject Achievement Score (T) × Once or Twice a Week	Subject Achievement Score (T) × Almost Every Day
**0**	**Reading**	0.47	−0.04	−0.75 *	−0.01	−0.02	−0.01
	**Math**	0.00	−0.05 *	−0.91 **	0.01	0.03	0.00
	**Science**	−0.03	−0.12	0.04	0.02	0.02	0.03
**1**	**Reading**	0.23	−0.42	−0.49 *	−0.00	0.03 *	0.01
	**Math**	0.33	−0.09	−0.39	0.00	−0.00	−0.00
	**Science**	0.10	−0.17	−0.02	−0.02	−0.00	0.01
**2**	**Reading**	−0.07	−0.26	−0.08	0.01	0.01	0.03 **
	**Math**	0.26	−0.04	−0.07	−0.00	0.01	0.03 *
	**Science**	−0.05	−0.14	−0.14	0.00	0.02	0.01

** *p* < 0.01; * *p* < 0.05. Shading has been added to statistically significant estimates. Cluster-robust standard errors are calculated with the cluster variable S2_ID when **T** = 0; S4_ID when **T** = 1; and S6_ID when **T** = 2.

## Data Availability

ECLS-K:2011 is an open-source dataset provided by the National Center for Education Statistics: https://nces.ed.gov/ecls/kindergarten2011.asp (accessed on 31 January 2022).
